# Supplementary material for: Initial Trans-Arterial Chemo-Embolisation (TACE) Is Associated with Similar Survival Outcomes as Compared to Upfront Percutaneous Ablation Allowing for Follow-Up Treatment in Those with Single Hepatocellular Carcinoma (HCC) ≤ 3 cm: Results of a Real-World Propensity-Matched Multi-Centre Australian Cohort Study
Source: Cancers (Basel). 2024 Aug 29;16(17):3010. doi: 10.3390/cancers16173010 (PMC11394053; doi:10.3390/cancers16173010)

## Slide 1
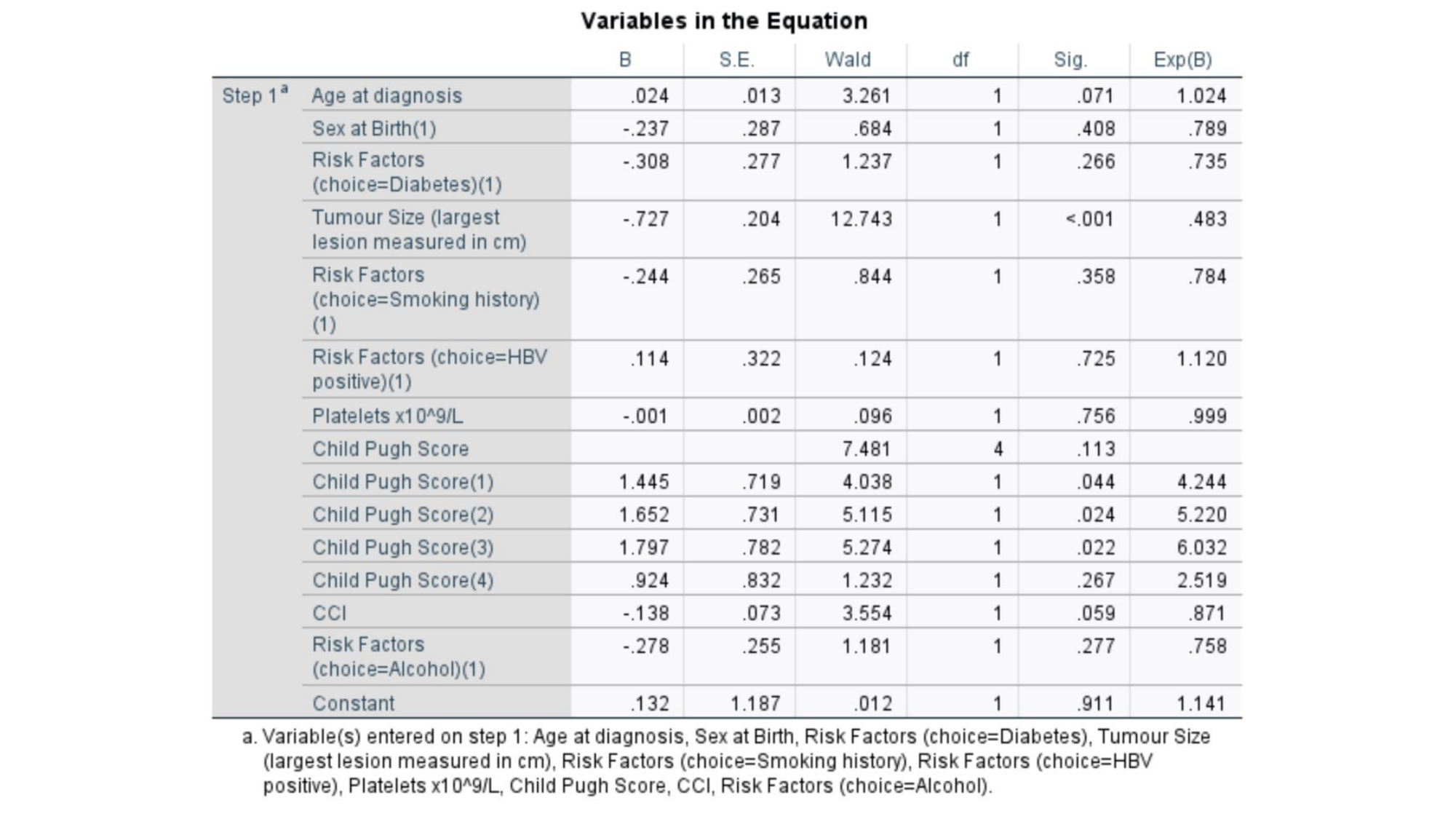

## Slide 2
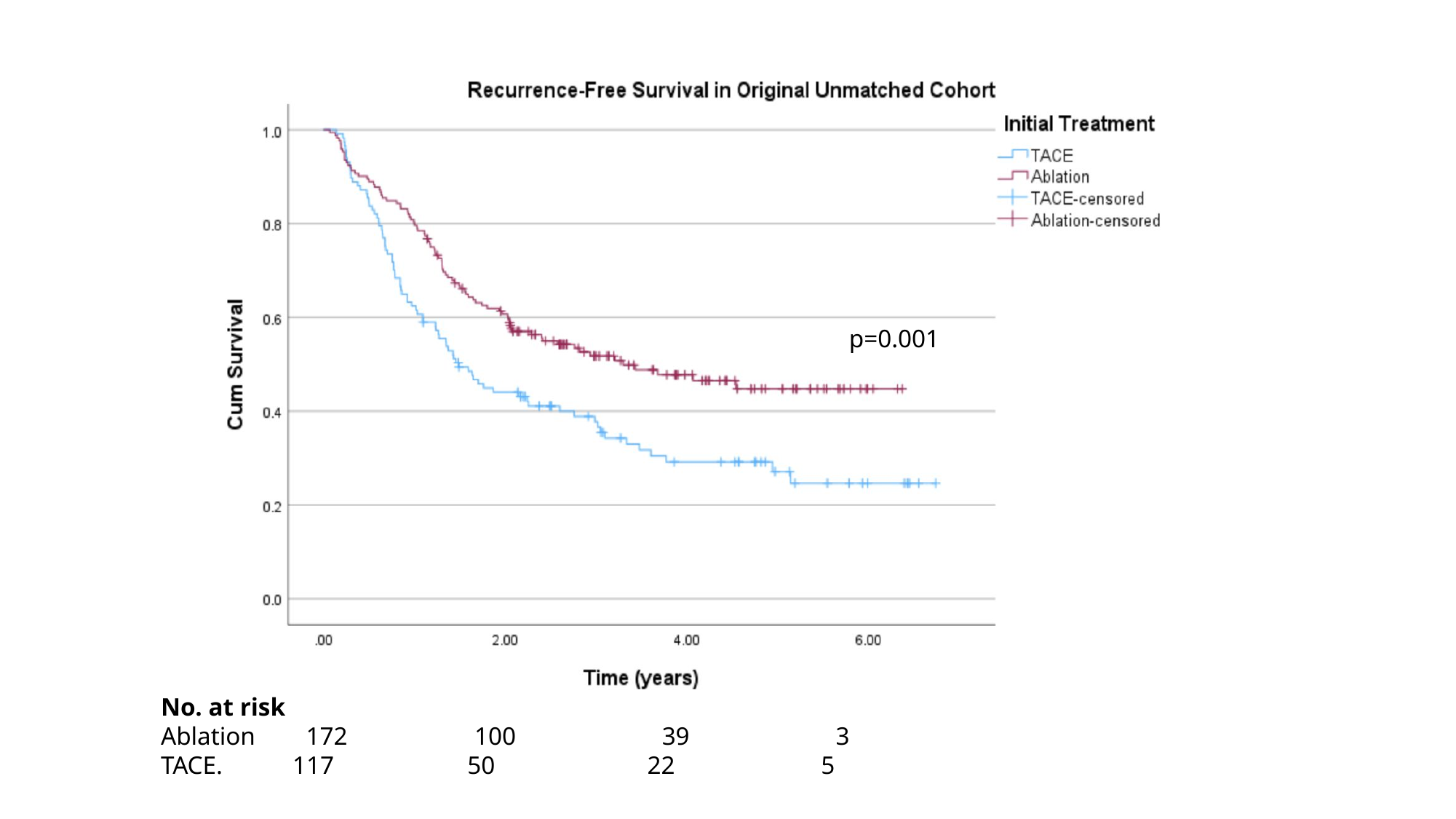

p=0.001
No. at risk
Ablation 172 100 39 3
TACE. 117 50 22 5

## Slide 3
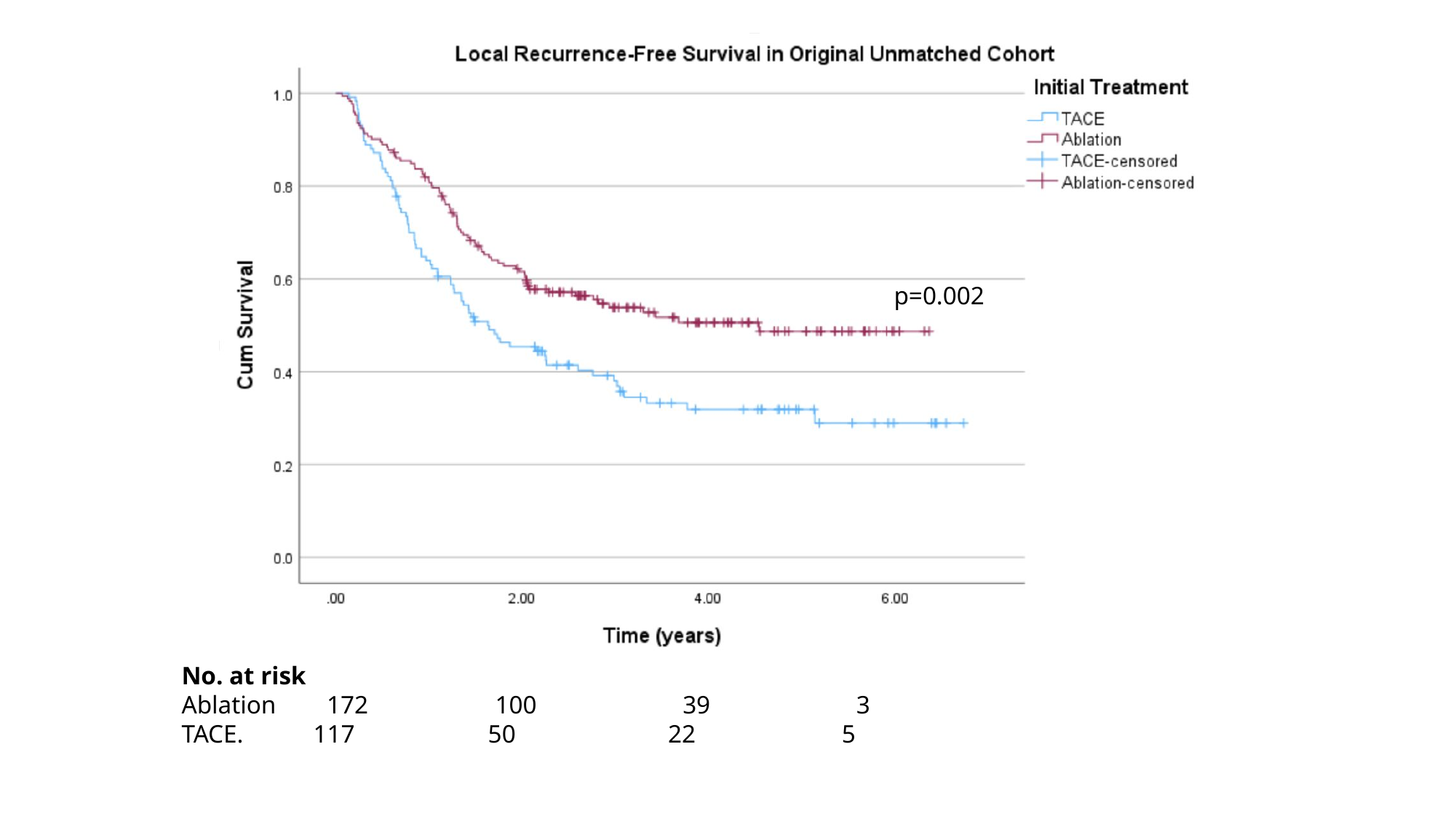

p=0.002
No. at risk
Ablation 172 100 39 3
TACE. 117 50 22 5

## Slide 4
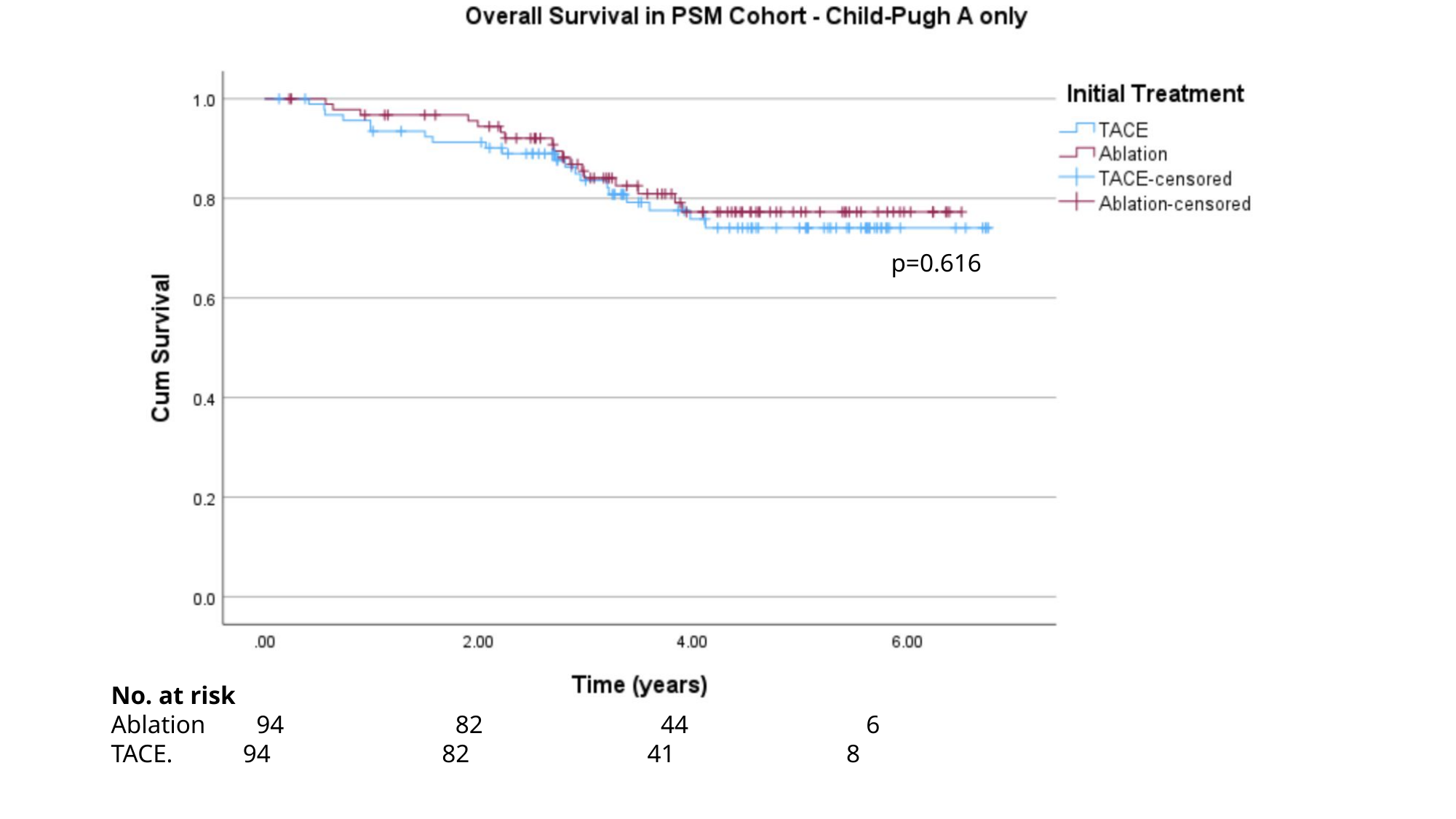

p=0.616
No. at risk
Ablation 94 82 44 6
TACE. 94 82 41 8

## Slide 5
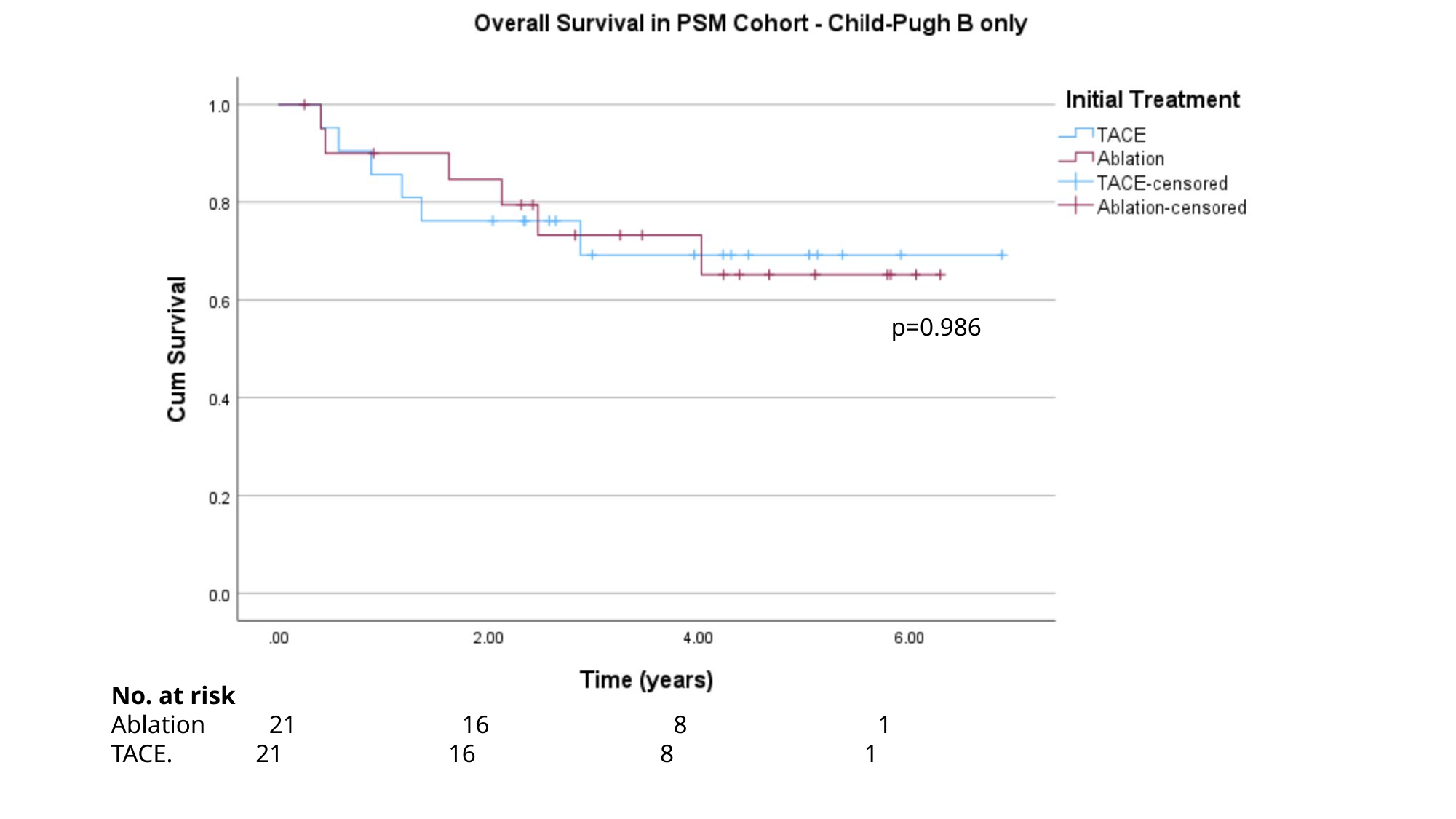

p=0.986
No. at risk
Ablation 21 16 8 1
TACE. 21 16 8 1

## Slide 6
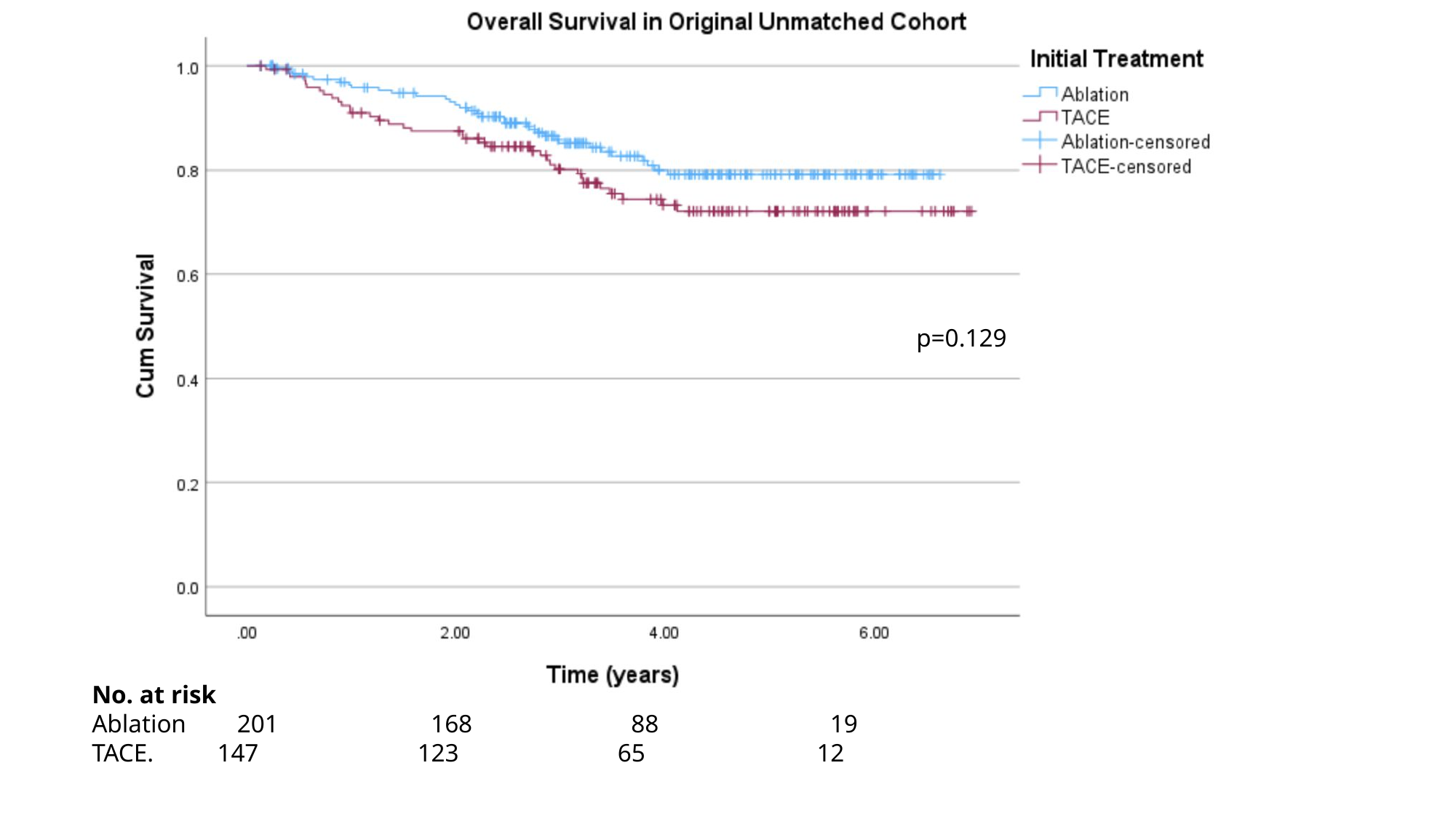

p=0.129
No. at risk
Ablation 201 168 88 19
TACE. 147 123 65 12

## Slide 7
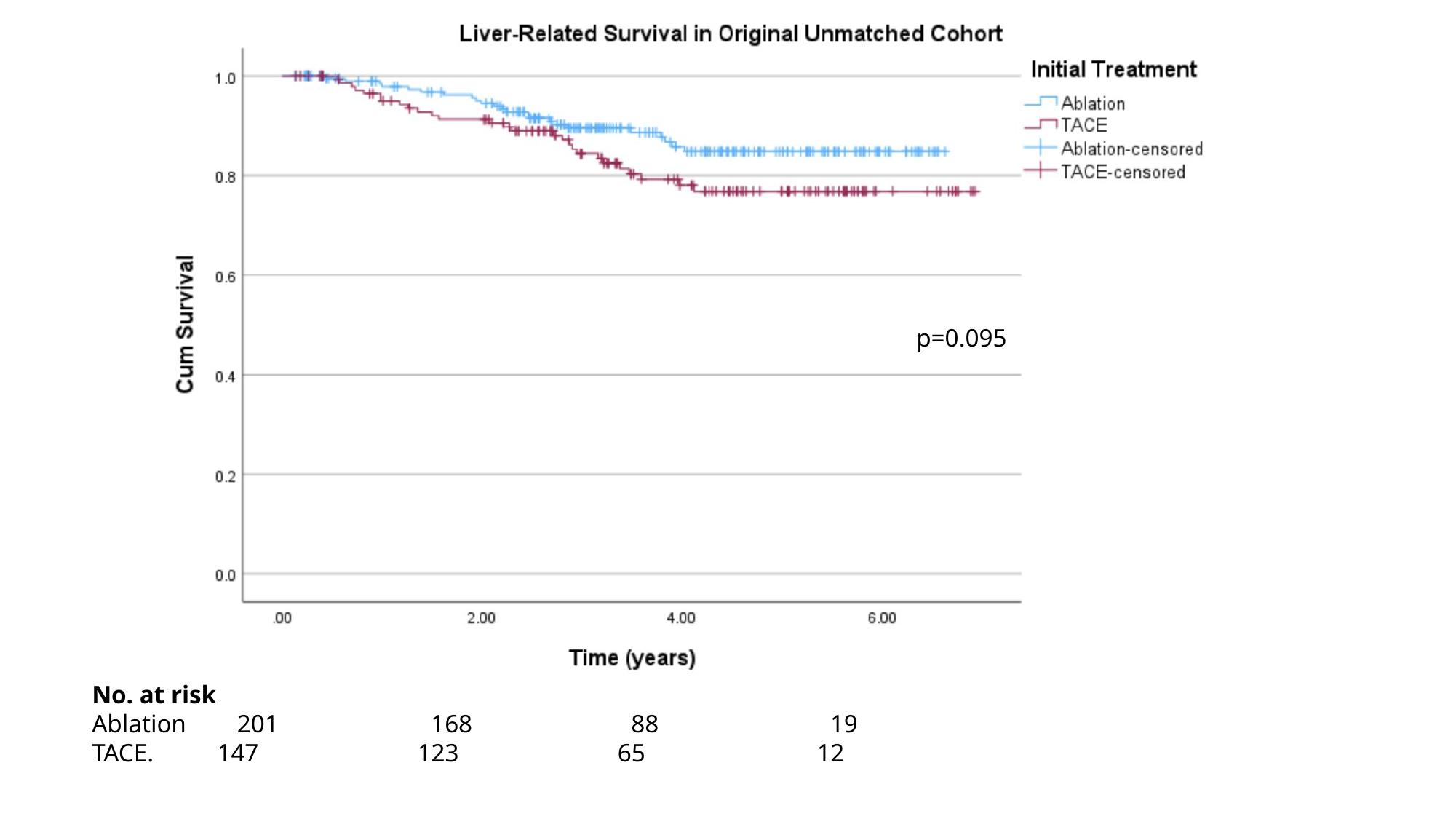

p=0.095
No. at risk
Ablation 201 168 88 19
TACE. 147 123 65 12

## Slide 8
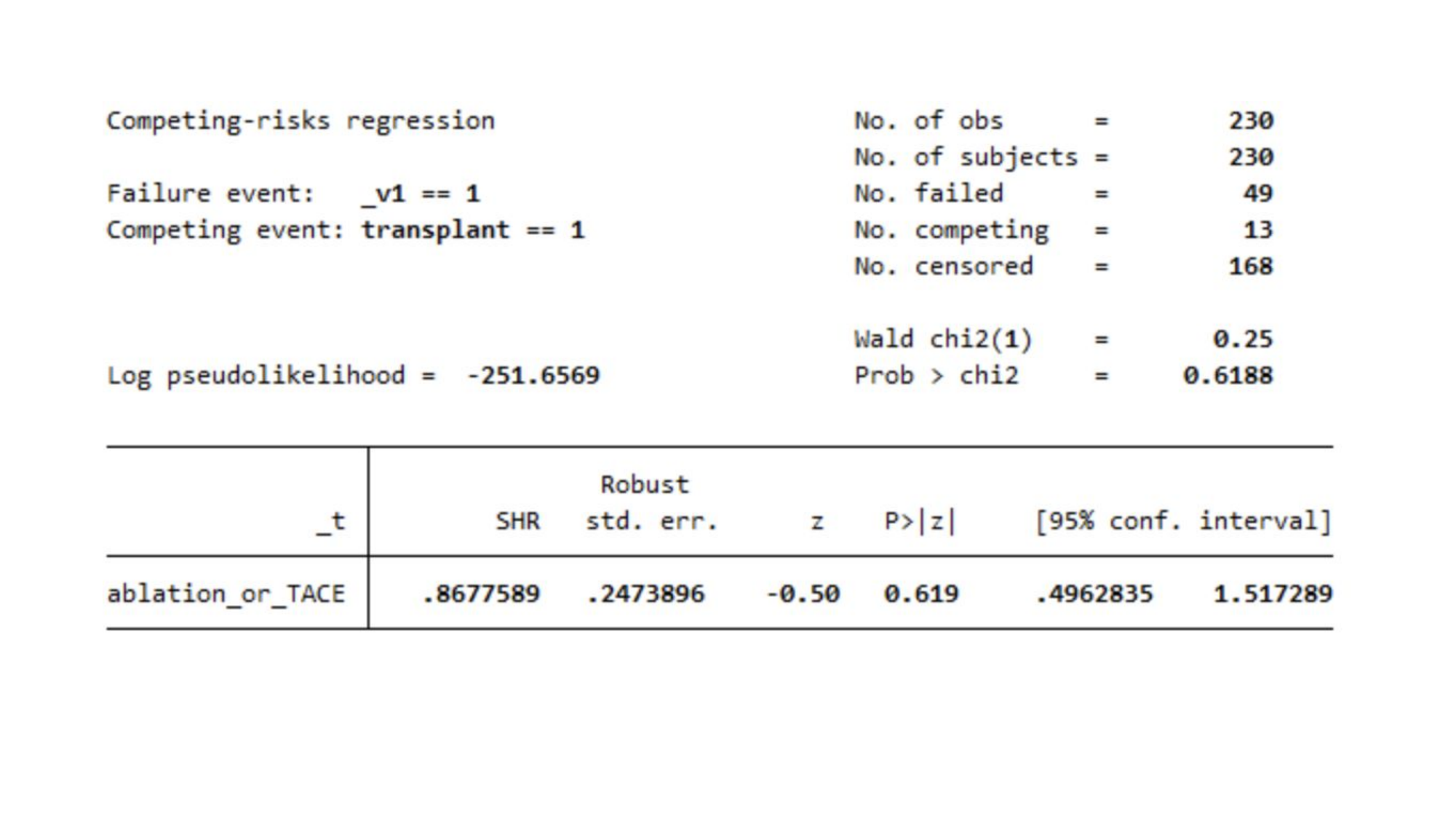

## Slide 9
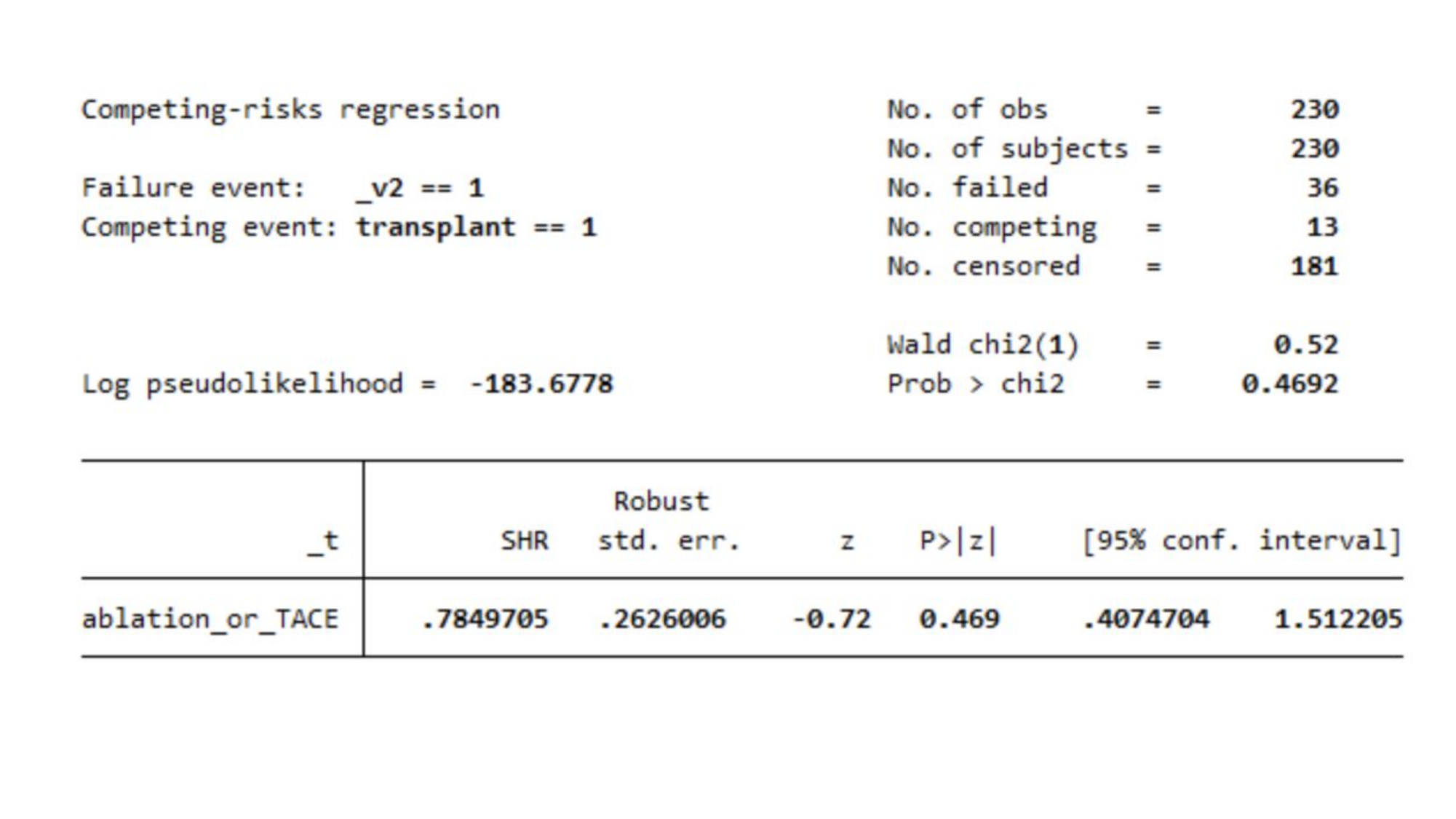

Supplement: Supplementary file 1 [file cancers-16-03010-s001.zip › cancers-3160593-supplementary.pptx]
